# Identifying youth-friendly service practices associated with adolescents’ use of reproductive healthcare services in post-conflict Burundi: a cross-sectional study

**DOI:** 10.1186/s12942-016-0075-3

**Published:** 2017-01-13

**Authors:** Imelda K. Moise, Jaclyn F. Verity, Joseph Kangmennaang

**Affiliations:** 1 Department of Geography and Regional Studies, University of Miami, 1300 Campo Sano Ave, Coral Gables, FL 33124 USA; 2 University of Waterloo, Waterloo, ON N2L 3G1 Canada

## Abstract

**Background:**

Very little is known about reproductive health service (RHS) availability and adolescents’ use of these services in post-conflict settings. Such information is crucial for targeted community interventions that aim to improve quality delivery of RHS and outcomes in post-conflict settings. The objectives of this study therefore was to examine the density of RHS availability; assess spatial patterns of RHC facilities; and identify youth-friendly practices associated with adolescents’ use of services in post-conflict Burundi.

**Methods:**

A cross-sectional survey was conducted from a full census of all facilities (n = 892) and provider interviews in Burundi. Surveyed facilities included all public, private, religious and community association owned-centers and hospitals. At each facility efforts were made to interview the officer-in-charge and a group of his/her staff. We applied both geospatial and non-spatial analyses, to examine the density of RHS availability and density, and to explore the association between youth-friendly practices and adolescents’ use of RHS in post-conflict Burundi.

**Results:**

High spatial patterning of distances of RHC facilities was observed, with facilities clustered predominantly in districts exhibiting persistent violence. But, use of services remained undeterred. We further found a stronger association between use of RHS and facility and programming characteristics. Community outreach, designated check-in/exam rooms, educational materials (posters, print, and pictures) in waiting rooms, privacy and confidentiality were significantly associated with adolescents’ use of RHS across all facility types. Cost was associated with use only at religious facilities and youth involvement at private facilities. No significant association was found between provider characteristics and use of RHS at any facility.

**Conclusions:**

Our findings indicate the need to improve youth-friendly service practices in the provision of RHS to adolescents in Burundi and suggest that current approaches to provider training may not be adequate for improving these vital practices. Our mixed methods approach and results are generalizable to other countries and post-conflict settings. In post-conflict settings, the methods can be used to identify service availability and spatial patterns of RHC facilities to plan for targeted service interventions, to increase demand and uptake of services by youth and young adults.

## Background

Access to reproductive health services (RHS) and information and by sexually active young people is fundamental to preventing unwanted pregnancies, managing rapid population growth and improving the health and economic well-being of families and communities [1–4]. The need for reproductive health information and services is particularly dire in post-conflict settings, where the capacity to provide health services, including for sexual and reproductive health, is often limited [5–7]. In these settings, healthcare systems are fragmented and siloed, trained healthcare providers are wanting and contraceptive supply chain systems in disarray. The result is low access to RHS, increases in unwanted teen pregnancies and sexual transmitted infections (STIs) [8]. For example, in Tanzania high births rates (30%) were documented among Congolese teens(ages 14 and 18 years) [9], and in Colombia displaced young girls aged 13–19 accounted for 30% of all births in 2000 [10]. A lack of basic reproductive health information was documented among adolescents in Nepal [11]. Together, these studies highlight the need for reliable information, counseling and tailored RHS for adolescents in conflict settings.

Research to date has documented a variety of youth-friendly service (YFS) practices that are positively and negatively associated with the provision of family planning (FP) and RHS [12]. YFS practices include provider characteristics (e.g., specially trained staff, ensuring privacy and confidentiality), as well as facility characteristics, such as, convenient wait times, operating hours, locations, and maintaining comfortable surroundings. Program design characteristics include affordable cost, having a wide range of services available, teen involvement in the design and in needs identification, short wait times and provision of timely referrals [13]. The provision of YFS even after adoption of effective approaches and strategies is more likely to vary across regions, such that high and low levels of service availability and adolescents’ use of FP/RH services including use of modern contraceptive methods are concentrated in specific geographic areas. Likewise, the extent to which characteristics of YFS are associated with adolescents’ actual use of reproductive healthcare (RHC) services is likely to vary across space.

Therefore, there is potential to use geospatial and non-spatial analyses to better understand the RHC service availability and adolescents’ use of these services. First, although research has been carried out on adolescent’s use of RHC services [13], no previous study has used fine-grained geographical administrative data from a census of facilities to assess RHC service availability and adolescents’ use of these services in post-conflict settings. Second, we are not aware of work that has systematically assessed whether associations between RHC service availability and areas of persistent violence vary across different geographic regions.

There remains much that we do not know about service availability and use of FP/RH services by adolescents in post-conflict settings. We have limited understanding of the ability of facilities to provide YFS to adolescents in these settings. Knowledge about the extent of RHC service availability and use of such services by adolescents in post-conflict settings could have important implications for tailoring interventions to specific communities based on the pertinent provider, facility and program design characteristics to increase uptake of FP/RH among these individuals. The objectives of this research are (1) to examine the density of RHC service availability, (2) to assess spatial patterns of RHC facilities, and (3) to identify YFS practices associated with adolescents’ use of RHC services in post-conflict Burundi. In our study, of RHC services will be used interchangeably with FP/RH services and include “family-planning, counseling services; prenatal and postnatal care and delivery; abortion services and post-abortion care; treatment and prevention of reproductive tract and sexually transmitted diseases and infections including HIV; and information and counseling about human sexuality [14].”

## Methods

### Study setting

The study was conducted in Burundi, an east-central African country. Burundi has an estimated population of 10.16 million as of 2013 and is one of the most densely populated countries in central Africa [15]. Although fertility rates have fallen over time, adolescent birth rates remain high. In 2011, there were 185 reported births per 1000 women aged 15–19 years [16].

**Fig. 1:**
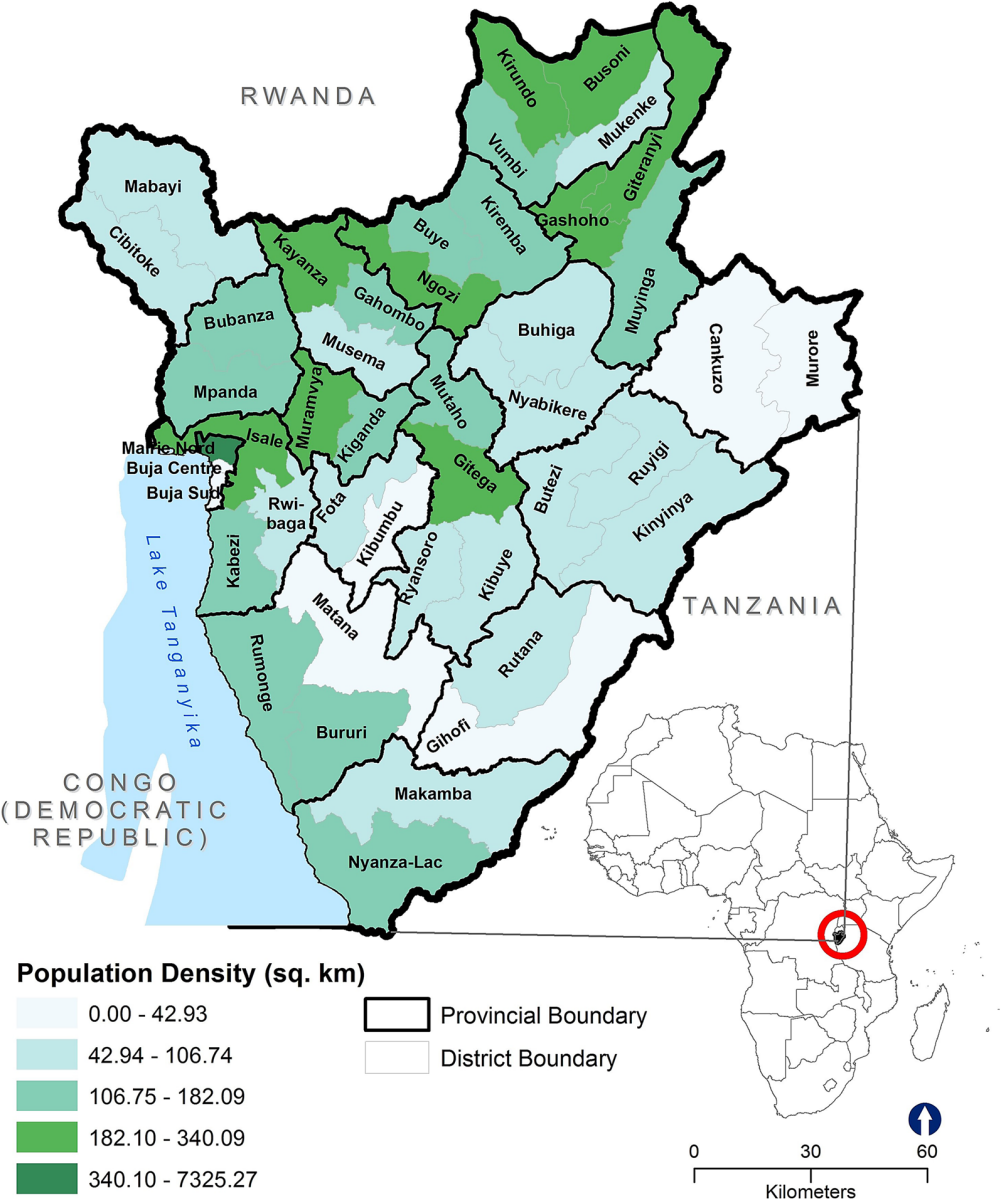
Urbanization levels and population in Burundi’s 46 districts

Nearly 90% of Burundi’s population lives in rural areas. Unfortunately, the country has been in repeated conflict since its independence in 1962. Years of continuous conflict has had an impact on the country’s population such that 65% of its population is comprised of young people. Figure 1 provides a general overview of the extent of urban and rural areas in Burundi.

### The Burundian healthcare system

Prolonged conflict has led to significant destruction and disruption of the healthcare system, services and quality of health. At the same time the government’s capacity to invest in the health sector is limited—in 2005, just 2.7% of the total budget was dedicated to health services [17]. Ongoing insecurity in certain regions has increased the inaccessibility of healthcare for many Burundians [18]. Access to healthcare was made more difficult for many by the adoption of a cost recovery system in 2002, where all patients regardless of their socio-economic status were required to pay for all medical costs and medicines. However, in 2004, the government of Burundi institutionalized output-based financial support or performance-based financing (PBF) of the facilities.

Alike neighboring Rwanda, the roll-out of PBF to Burundi’s 18 provinces between 2006 and 2010 has been seen as a way to improve quality of care, coverage of services, strengthen local facilities and to remove user fees in the health sector to children under five and pregnant women. Facilities are free to assign their finances to different uses according to their needs (e.g., day-to-day operations, invest in small equipment, staff incentives). Likewise, primary care is provided by private and publicly operated facilities. Facilities are contracted and partially funded on the basis of their performance by a third-party agency independent of providers [i.e., NGO or Ministry of Health (MoH)]. The third-party agency is responsible for evaluating performance and providing subsidies and acting both as a purchaser and an inspector. Every time a facility (contractor) delivers a contracted service, it is eligible for a unit subsidy. Notably, more than half of the contracted indicators at the facility-level have been services for which users are not required to pay any fee.

## Study design

**Fig. 2:**
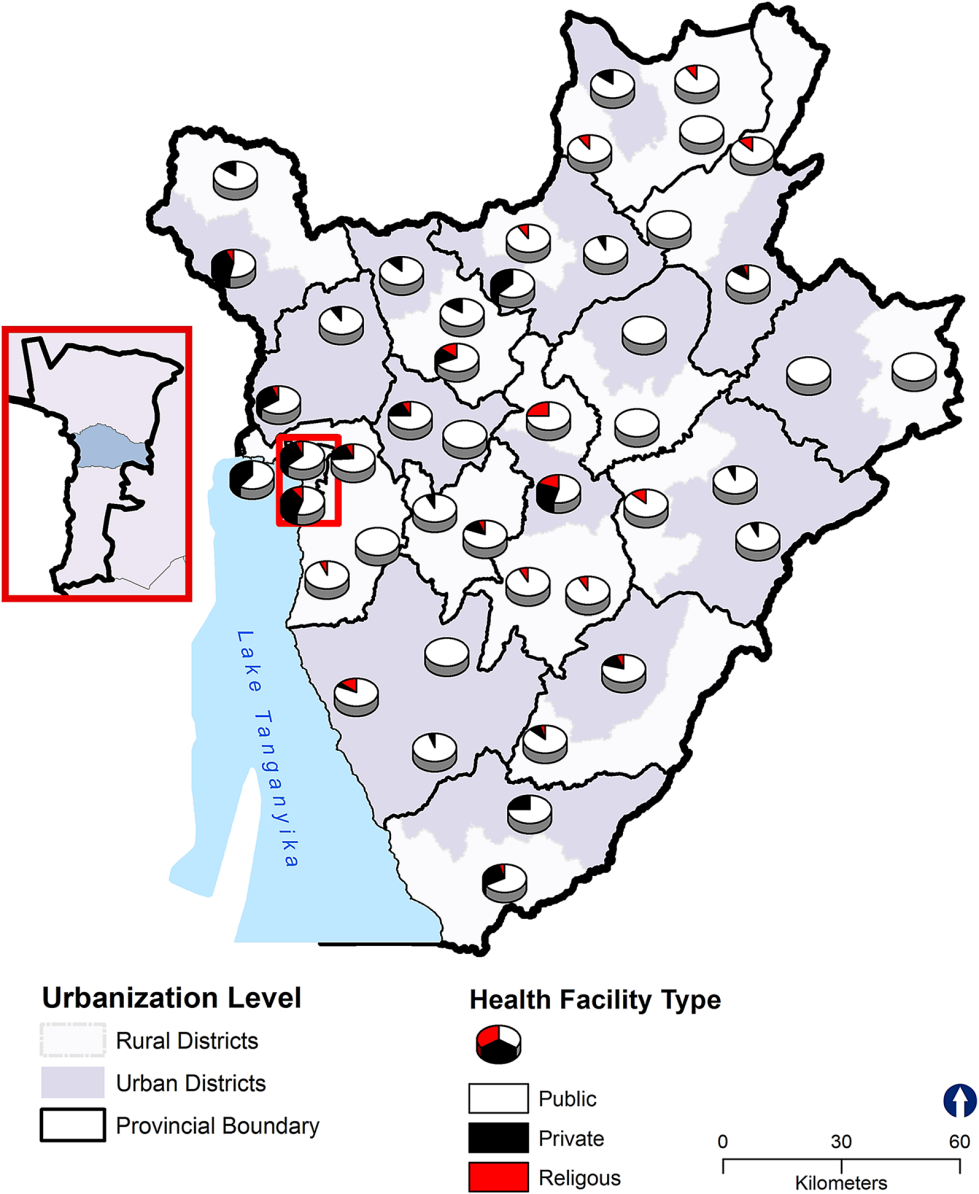
The spatial distribution of all healthy facilities in Burundi by facility type and urbanization level

We examined facility-based RHC services using data from the 2013 Population Services International (PSI) Burundi’s Sexual and Reproductive Health Survey (SRHS). This cross-sectional survey was designed to collect information on the landscape of sexual and reproductive health services available for adolescents across health facilities in the country [19]. PSI conducted a full census of all facilities (n = 892), including public (n=538), private (n=195), religious (n=139) and community association (n=20) owned-facilities and hospitals. At each facility efforts were made to interview the officer-in-charge and a group of his/her staff. Figure 2 shows the spatial distribution of all facilities in Burundi by facility type and urbanization level.

### Health facilities data source

The 2013 SRHS data included observation data from facilities and interviews with providers. The observation data assessed the extent to which facilities practices followed standards of care that are generally recommended for the provision of YFS. Trained staff observed adolescents’ visits, the use of procedures; examinations conducted, documented the availability of commodities, and discussed usual RHC practices with providers on site. This information was cross checked by reviewing records (e.g., registers, logbooks and monthly reports submitted to the MoH) for each facility. Interviews with providers were conducted face-to-face in the facilities to assess facility, provider and program design characteristics [19].

## Measures

### Response variable

Our response variable is continuous (the number of youth and young adults who visited each facility in the past 7 days for a RHC service). This variable assesses youth and young adults’ use of RHS. During the survey, trained staff reviewed facilities records and noted the number of adolescents who visited each facility. Adolescents were defined as persons aged between 10 and 24 years both male and female, a definition also applied in related studies [13]. Demographic information such as gender and marital status was recorded at the aggregate-level.

### Independent variables

We assessed facilities characteristics, measures of provider practices and program design factors. Facility characteristics included accessibility and environmental adaptation factors. We have chosen to group facilities into two categories (hospitals and health centers), with facility ownership (public, private, religious) used as the form of indicator variable for ‘facilities’.

To assess training, providers indicated whether or not they had received specific in-service or pre-service training in relation to the care of youth and young adults and training relating to meeting the special needs of teens. Providers were also asked to indicate whether peer educators (other youth or young adults) are involved in promoting the facility’s services and activities. Two other questions asked providers to indicate whether they have been informed of the rights of young people and if all providers at each facility know at least five of those rights.

To examine program design characteristics, providers reported whether or not the facility is involved in outreach efforts, has partnerships with community organizations and other sectors (e.g., schools, NGOs) to reach young people, and to indicate whether or not patient medical records are preserved to protect the privacy and confidentiality of young people. The provided facility, provider and program design information was cross checked by observations and by reviewing records, registers and logbooks for each facility. Additionally, respondents indicated whether or not the facility has educational materials and contracts for PBF.

To estimate contraceptive modern methods availability, trained staff conducted physical inventory counts, reviewed stock cards, quarterly management reports on contraceptive products and recorded the number of different types of contraceptive methods available (e.g., implants, emergency contraceptives, IUDs, injectables, condoms and pills). A contraceptive stock-out was considered if it was offered by the facility but not available for any part of the past 3 months. Table 1 shows the frequencies of variables used in the current study.

**Table 1:** Means and frequencies for all variables included in the study across Burundi, 2013

	Total N	N/(%)
Provider characteristics		
Specially trained staff		
Staff have received training in relation to the care of adolescents’ RH	872	19 (2.2)
Staff have received training to meet adolescents’ special RH needs	872	22 (2.5)
Facility uses peer educators/counselors	874	18 (2.1)
Ability to relate to youth in a respectful manner		
Staff have been informed of the rights of adolescents	874	92 (10.5)
All staff know at least 5 of the rights of adolescents	872	76 (8.7)
Health facility characteristics		
Accessibility		
Dedicated adolescent only hours and/or days	858	34 (3.8)
Facility hours includes evenings and/or weekend hours	865	674 (78.0)
Environmental adaptation		
Designated adolescent check-in rooms available	858	679 (79.0)
Waiting and exam rooms have pictures to appeal to adolescents	873	118 (13.5)
Waiting and exam rooms have print materials to appeal to adolescents	560	297 (53.0)
Waiting and exam rooms have posters to appeal to adolescents	557	320 (57.5)
Program design characteristics		
Adolescents are involved in the design and continuing feedback	871	16 (1.8)
Facility has a strategy to involve adolescents in planning and care provision	873	38 (4.4)
RHC services discounted to adolescents	464	52 (11.2)
Outreach		
Facility has partnerships with community organizations and other sectors, e.g., schools, NGOs to reach young people	858	94 (11.0)
Outreach and/or education provided in the community for young people	768	91 (11.8)
Facility has sign outside that states that all adolescents are welcome	516	7 (1.4)
Confidentiality		
Records are preserved to protect the privacy and confidentiality of adolescents’ personal medical records and health information	868	620 (71.4)

Pictures, posters and print materials include educational materials or information relating reproductive health

*RH* Reproductive health, *RHC* Reproductive Health Care

### Violence and administrative boundary GIS data

At each facility, Global Positioning System (GPS) coordinates were collected to help in estimating the density of FP/RH service availability. We downloaded conflict and protest data from the Armed Conflict Location and Event Data (ACLED) project [20]. Variables in the dataset include the type of conflict, the coordinates of the incidences, estimated fatalities, warring groups, among other factors. Data from 1997 to 2013 were used to locate clusters of persistent conflict. We obtained administrative boundaries (shapefiles) districts (n = 46) and provinces (n = 18) from the Directorate of the Burundi National System of Health Information.

### Geospatial analysis

#### Kernel density estimation and spatial proximity

We were interested in the density of RHC service availability and discrepancies in use of these services by adolescents at the facility-level unit of analysis. Therefore, we utilized geolocation facility data, and employed kernel density estimation (KDE) using the Spatial Analyst tools feature in ArcGIS version 10.3.1 [21] to estimate access to FP/RH services. The density of RHC service availability has been found to be a good proxy for access to FP/RH services than other measures of accessibility such as Euclidean distance [22].

KDE is characterized by the degree to which geographically close points (RHC facilities) that are at the center of the radius tend to be weighted higher than facilities at the margin [22, 23]. We created density variables by converting all of the geolocation facility data for each RHC facility, conflict and protest data (latitude and longitude) into continuous surfaces. This estimate gives a formal indication of areas of high RHC service availability (facilities and violent events per square kilometer) or high service use and high violence areas; and areas of low service and low violence [24]. KDE requires the user to specify the choice of circle radius. In this study, we use a radius of 4 km to represent an hour of travel time by foot as used in a previous study [22].

To measure proximity to facilities, we applied a buffer analysis. As with KDE analysis above, we used a buffer zone with radius of 4 km around all RHC facilities in Burundi. This radius reflects the localized nature of facility use in Burundi.

#### Average nearest neighbor

The Average Nearest Neighbor tool was used to assess the spatial patterning of distances among RHC facilities in the country. The tools allowed us to measure the degree to which RHC facility locations cluster or are spatially near to each other. This measure gives a formal indication of clustering and dispersion [25]. Generally, the average nearest neighbor tool returns values in the final output, the “Nearest Neighbor Index (NNI)” which denotes the quotient of the Observed Mean Distance to the Expected Mean Distance. In particular, a value less than 1 suggests that the distribution pattern of the RHC facilities in Burundi is clustered, and if the index is more than 1, the trend is toward dispersion. In other words, shorter distances than would be expected under spatial randomness are interpreted as clustering, whereas longer distances are interpreted as dispersion. All geospatial analysis was done in ArcGIS, version 10.3.1 [21].

### Statistical analysis

We generated descriptive statistics to describe the location of facilities, the availability of modern contraceptive methods, adolescents’ use of RHS, and to describe the existence of PBF contracts in these types of care. We then investigated the associations between our response variable and the independent variables with multivariable regression models. Because our response variable is recorded as a count (the number of adolescents who visited a RHC facility for a RH service in the past 7 days), we employed a Poisson regression. Moreover, since the response variable is not normally distributed, multiple linear or logistic regressions are impossible.

Notably, the Poisson model assumes an infinitely large population from which counts are drawn, and in the case of this study, the size of the adolescent population in Burundi is large relative to the number of reported counts. Hence, the Poisson distribution can be used as an approximation to the binomial distribution, since the Poisson mean >0. In our study, the Poisson regression model expresses the log outcome rate (use of RHC services) as a linear function of a set of selected youth-friendly practices at each facility. We fit parsimonious models by removing variables one at a time (non-significant variables), beginning with the one with the largest *P* value until all variables included in the model were significant (*P*  <  0.05). Table 3 presents the retained variables used in the study. The ‘health facility’ and PBF contractor as variables are used as indicator variables (e.g., Public = 1; Private = 2; Religious = 3; PBF contractor—0 = no, 1 = yes) in the regression analysis. All analyses were completed with IBM SPSS Statistics for Windows, Version 22.0 [26].

## Results

### Descriptive summary

A total of 24,232 adolescents aged 10–24 years visited RHC facilities in Burundi in the past 7 days prior to the survey (Table 2). Of the adolescents, 11% (2542) were male and 89% (20,821) were female, with an average gender sex ratio of 12:100. Married adolescents (72%, 16,488) were more likely to use RHC services than single adolescents (29%, 6587).

**Table 2:** Distribution of health facilities according adolescent demographics and whether a facility offers RHC services by facility ownership, Burundi, 2013

	Facility ownership (n = 892^a^)			
	Public N (%)	Private N (%)	Religious N (%)	Total N
Centers	(n = 495)	(n = 205)	(n = 125)	
Married, 10–24 years	11,130 (48.2)	1118 (4.8)	3172 (17.4)	15,420
Single, 10–24 years	4742 (20.6)	685 (3.0)	853 (13.7)	6280
Females, 10–24 years	14,371 (61.5)	1445 (6.2)	3730 (16.0)	19,546
Males, 10–24 years	1343 (5.7)	368 (1.6)	607 (2.6)	2318
Center offers RHC services	471 (57.0)	75 (9.1)	30 (3.6)	576
Center contracts with PBF	473 (54.6)	75 (8.7)	114 (13.1)	662
Center does not offer RHC services	12 (1.5)	101 (12.2)	81 (9.8)	194
% of visits in the past 7 days	67.1	3.4	18.7	
Average # of visits in the past 7 days	147.4	31.2	74.0	
Hospitals	(n = 43)	(n = 10)	(n = 14)	
Married 10–24 years	716 (3.1)	42 (0.2)	310 (1.3)	1068
Single 10–24 years	188 (0.8)	3 (0.0)	116 (0.5)	202
Females 10–24 years	888 (3.8)	58 (0.2)	329 (25.8)	1185
Males 10–24 years	165 (0.7)	2 (0.0)	57 (25.4)	224
Hospital offers RHC services	31 (3.8)	3 (0.4)	3 (0.4)	37
Hospital does not offer RHC services	9 (1.1)	3 (0.4)	7 (0.8)	19
Hospital contracts with PBF	42 (4.8)	5 (0.6)	11 (1.3)	58
% of visits in the past 7 days	4.6	4.6	1.6	
Average # of visits in the past 7 days	40.0	52.6	48.2	

The percentage (%) of adolescents’ visits in the past 7 days was calculated by dividing the total number of adolescents who visited facilities that offer RHC services by total visits

^a^66 health facilities had missing data

# Number

About 67% of all adolescents’ visits were to public RHC facilities, for an average number of visits in the past 7 days of 147. Less than 5% adolescents visited hospitals and private centers. Additionally, of the 613 RHC facilities, most were public health centers or health posts (52.8%). Three-quarters of facilities (78%) were located in rural districts, and almost 85% of all facilities reported serving as PBF contractors.

**Fig. 3:**
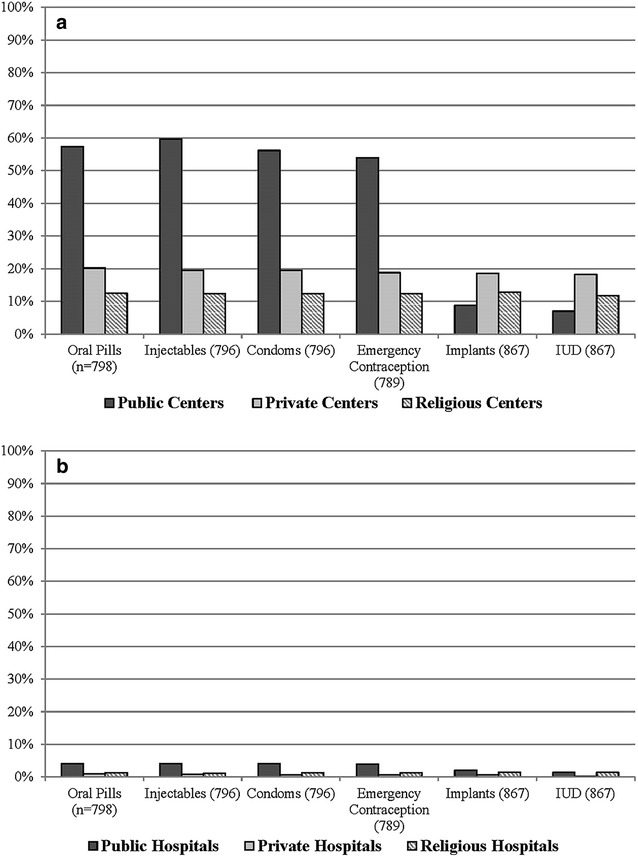
Percentage of all health centers (**a**) and hospitals (**b**) with no stock-outs within min-max level in the past three-months prior to the survey. *Note*: the “Min” value represents a stock level that triggers a reorder and the “Max” value represents a new targeted stock level following the reorder

More than 50% of public facilities reported no stock-outs of four modern contraceptive methods (oral pills, injectibles, condoms and contraception pills) at any point in the past 3-months prior to the survey. Only less than 20% of private and religious health centers reported no stock-outs of modern contraceptive methods during the same period (Fig. 3a). The availability of implants and IUDs is low across health centers regardless of facility-type. Hospitals (all ownership types) exhibited the highest stock-outs. Only 10% of hospitals reported no stock-outs of the six modern contraceptive methods (oral pills, injectibles, condoms, emergency contraception, implants and IUDs) (Fig. 3b).

### Estimating the density of RHC service availability, conflict and spatial proximity

**Fig. 4:**
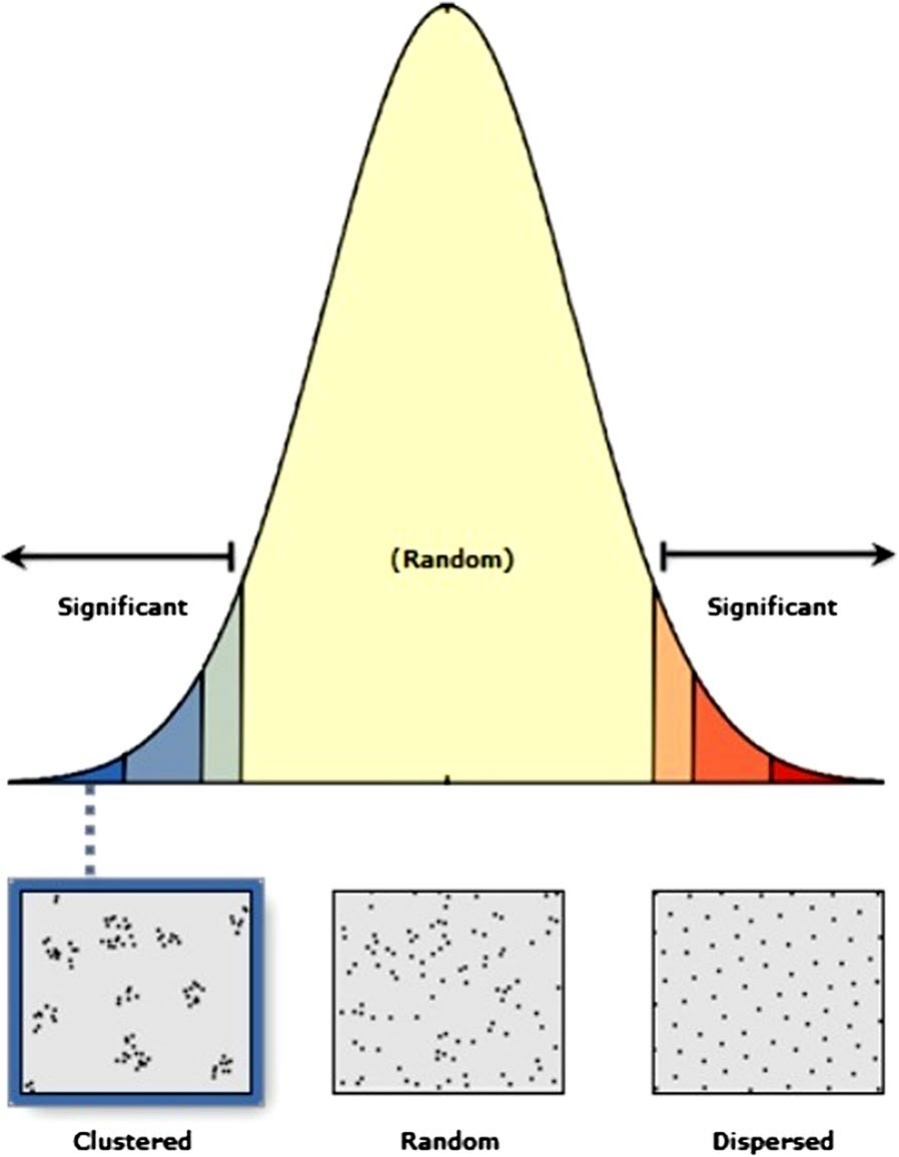
The results of average nearest neighbor for health facilities (centers and hospitals)

**Fig. 5:**
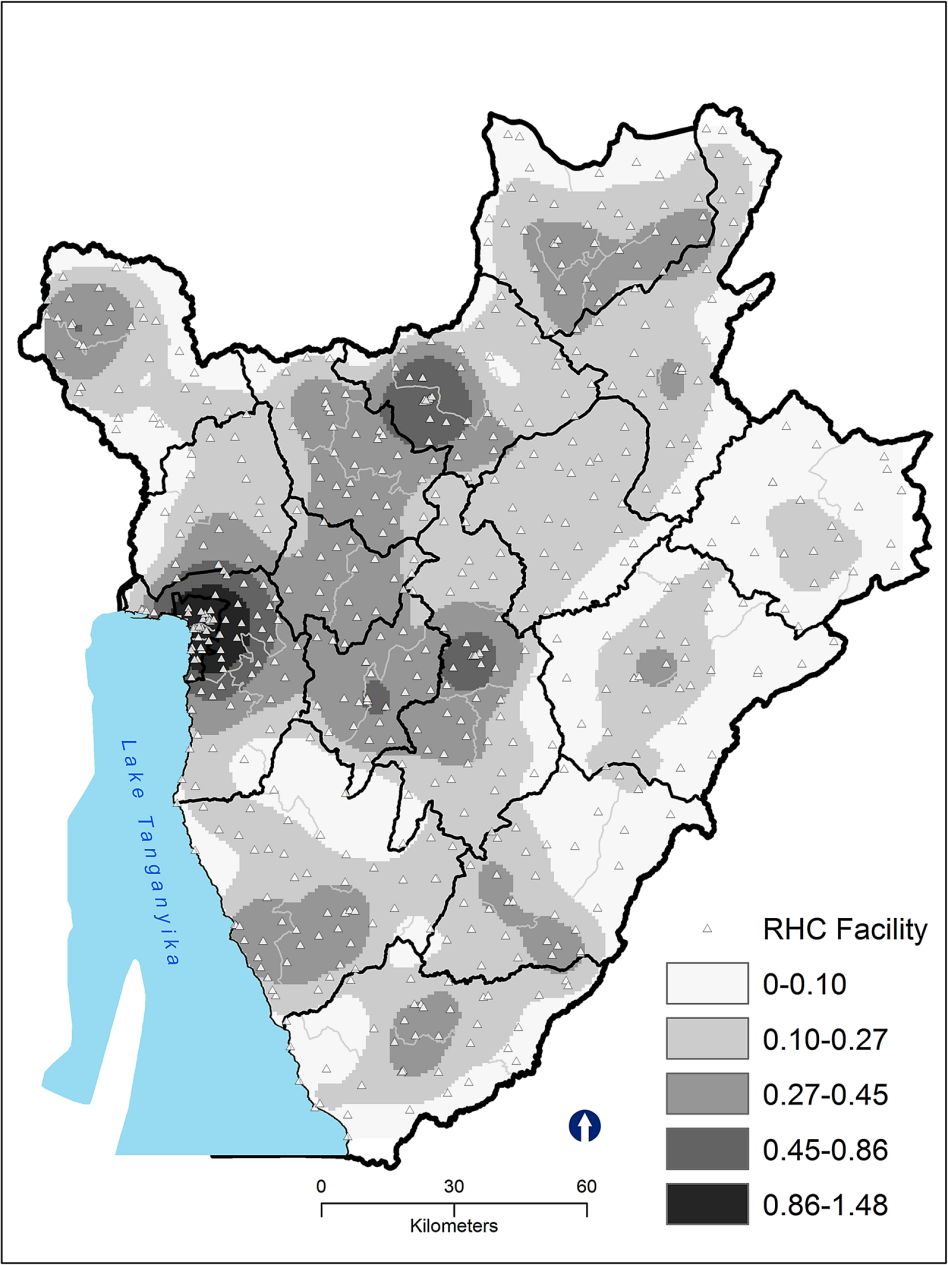
Density of reproductive health service facilities (facilities per square kilometer) according to kernel estimation: Burundi, 2013. The locations of reproductive health service facilities are marked by *white triangles*

We found high spatial patterning of distances of RHC facilities in Burundi (Z-score: −3.61, *P* value: .0001) (Fig. 4). Peaks were found within the 4 km radius, which represent an hour of travel time by foot. The high densities were concentrated in four districts located in two provinces of Bujumbura Mairie (Buja-Nord, Buja-Center and Buja-Sud) and Bujumbura Rural(Isale), followed by districts located in provinces of Ngozi, Kayanza, Cibitoke, Gitega, Mwaro and parts of Bururi (Fig. 5). Cankuzo, a rural province located in eastern Burundi had the lowest density of RHC facilities.

**Fig. 6:**
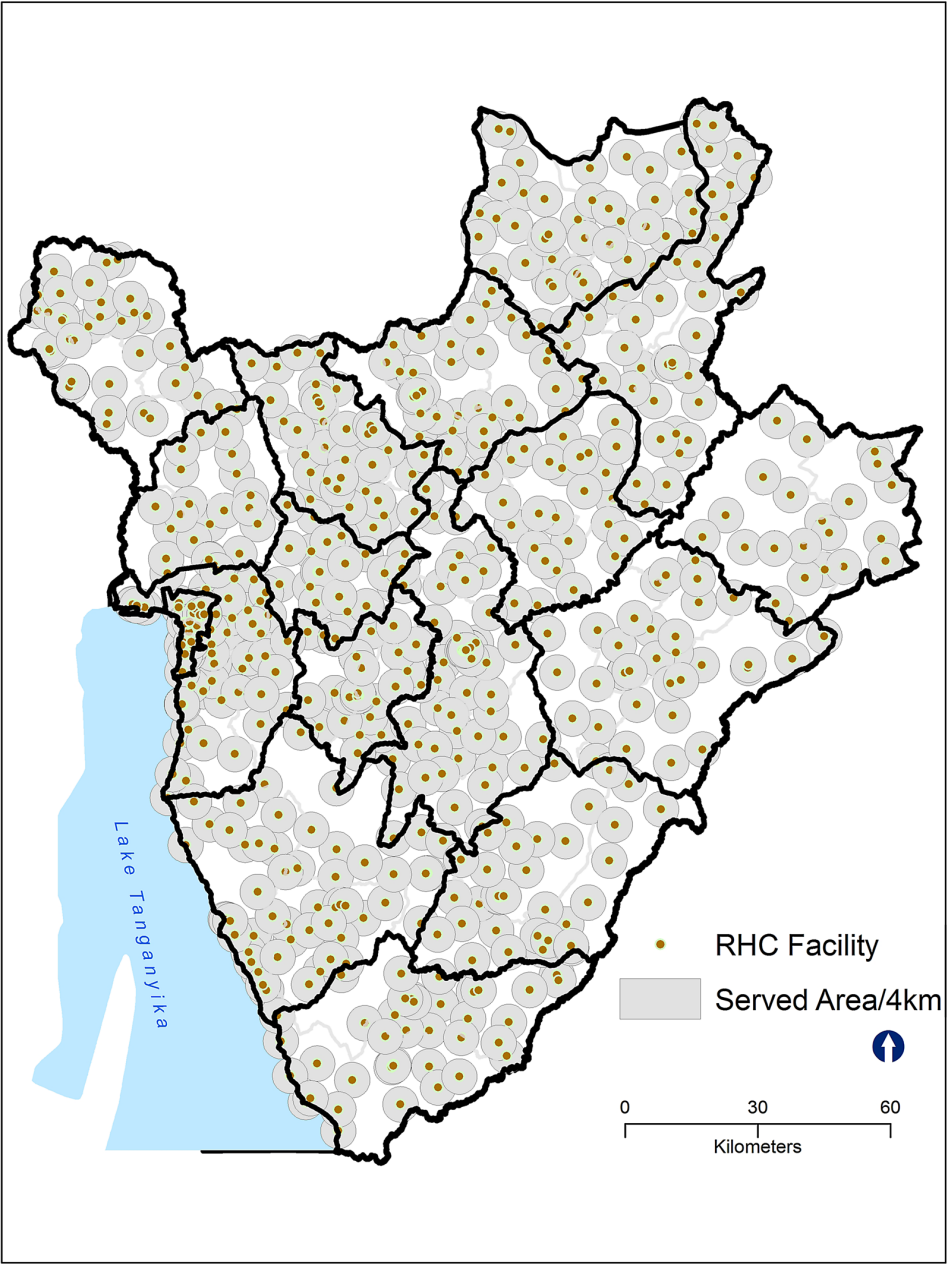
Density of areas of persistent violence (violent events per square kilometer) according to kernel estimation: Burundi, 2013. Adolescents’ use of services in these areas is denoted by graduated symbols in *dark green*

**Fig. 7:**
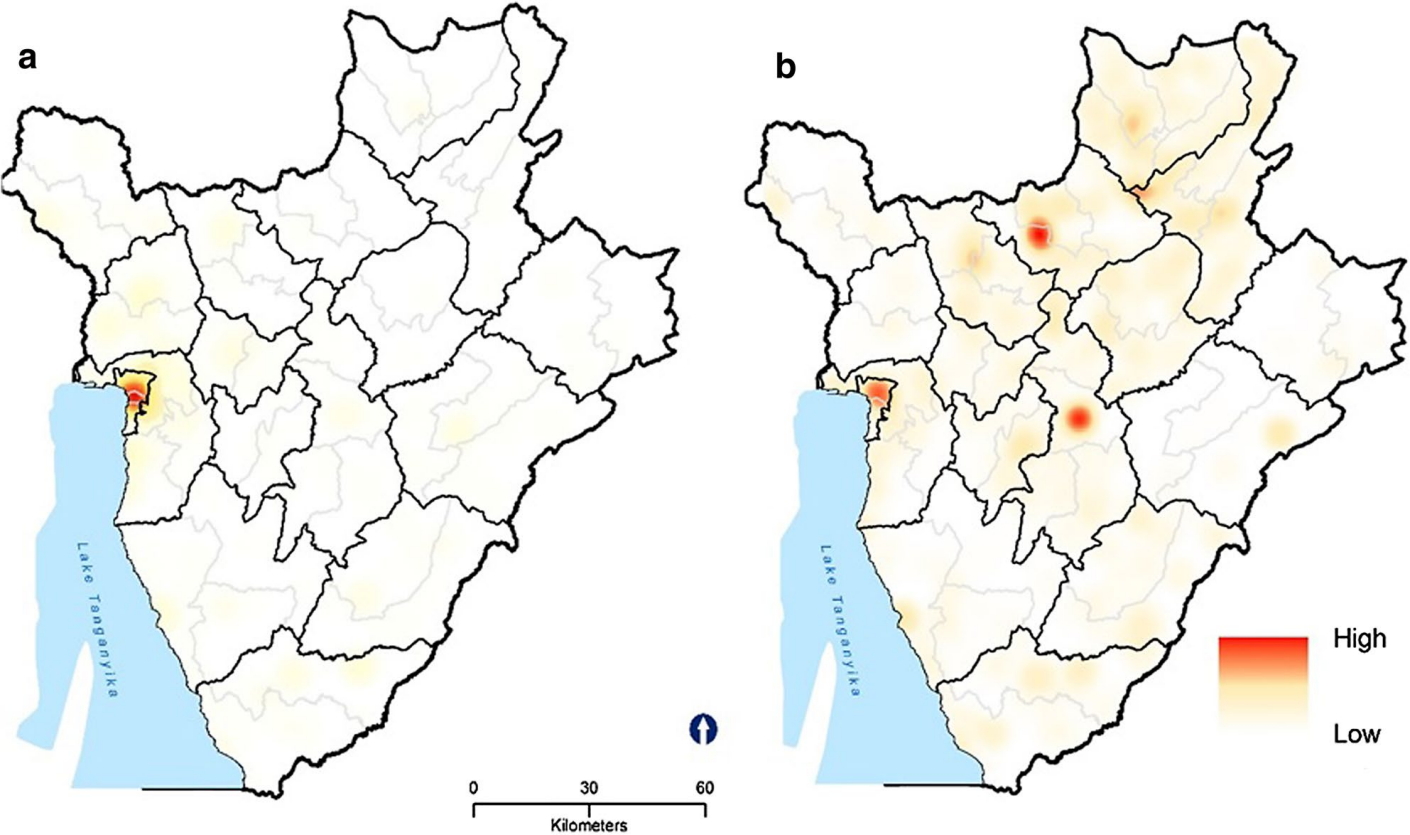
4-km reproductive healthcare service delivery points in Burundi

We identified the districts wherein there were higher densities of persistent violence. Using the 4 km radius, we found that three urban districts of Bujumbura Mairie province (Fig. 7) exhibited persistent violence (conflict, incidences, fatalities, and warring groups). Despite this, use of RHC services by adolescents is high in these districts (Fig. 6). However, we observed low use of RHC services in less affected districts located in Cankunzo, Ruyingi, Bururi, Cibitoke and Mwaro provinces. Almost all RHC facilities in Burundi are within the 4 km catchment zone, suggesting high RHC accessibility (Fig. 7).

### Youth-friendly service practices associated with use of reproductive health services

As the results in Table 3 indicate, the association between adolescents’ use of RHS and each YFS practice varied between facility types. Notably, facility and program design characteristics were both positively associated with RHS use among adolescents. Across all facility types, community outreach, having designated check-in and exam rooms, educational materials (posters, print and pictures) in waiting and exam rooms that appeal to adolescents, and privacy and confidentiality were significantly associated with adolescents’ use of services (*P* < 0.0001).

**Table 3:** Association of key YFS characteristics related to adolescents’ use of RHS using a Poisson regression model

Explanatory variables/youth-friendly practices	Public (n = 499)			Private (n = 215)			Religious (n = 139)		
	β	Wald 95% confidence limits		β	Wald 95% confidence limits		β	Wald 95% confidence limits	
		Confidence interval	Sig.		Confidence interval	Sig.		Confidence interval	Sig.
Intercept	94.298	80.89–109.92	***	0.005	0.005–0.005	***	26.754	17.17–41.68	***
Health facility characteristics									
Hours include evenings/weekend hours	0.739	0.698–0.781	***	1.281	0.999–1.643	*	0.824	0.704–0.965	*
Designated check-in and exam rooms available	0.113	0.068–0.187	***	.040	0.006–0.289	***	1.487	1.190–1.857	***
Waiting/exam rooms have print educational materials to appeal to adolescents	0.652	0.611–0.696	***	.124	0.057–0.271	***	0.625	0.53–0.736	***
Waiting/exam rooms have educational pictures to appeal to adolescents	0.805	0.758–0.855	***	2.819	1.822–4.363	***	0.513	0.440–0.597	***
Waiting/exam rooms have educational posters to appeal to adolescents	1.618	1.527–1.714	***	.680	0.490–0.944	*	1.445	1.273–1.640	***
Program design characteristics									
Adolescents are involved in design/feedback	~	~	~	.520	0.346–0.781	**	~	~	
Facility has strategy to involve adolescents^a^	0.939	0.838–1.052		1.970			2.663	1.983–3.576	***
Privacy and confidentiality preserved^b^	0.698	0.628–0.775	***	2.481	1.836–3.352	***	1.573	1.338–1.849	***
Outreach and/or education is provided^c^	0.889	0.841–0.939	***	.113	0.08–0.158	***	0.773	0.684–0.873	***
RHC services discounted to adolescents	1.079	0.970–1.200		~	~	**~**	1.399	1.069–1.830	*

*β* Exponential estimates

Significance level: <0.0001***; <0.01**; <0.05*

~ Model did not converge

^a^Facility has a strategy to involve adolescents in planning and in the provision of care

^b^Records are preserved to protect the privacy and confidentiality of adolescents’ personal medical records and health information

^c^Outreach and/or education provided in the community for young people

Discounted services were positively associated with use of RHS (*P* < 0.05). Among religious facilities, and we found no evidence that discounted services were associated with use of services at public and private facilities. The same is true for adolescents’ involvement in design and continuing feedback. The association is only with private facilities (*P* < 0.01). Provider characteristics were not statistically associated with the use of RHS at any facility type. Privacy and confidentiality, having educational pictures and posters in waiting and exam rooms to appeal to adolescents and having hours that include evenings and weekend hours were significantly associated with PBF status (Table 4).

**Table 4:** Association of key YFS characteristics related to performance-based financing status using a Poisson regression model

	β	Confidence interval	Sig.
Intercept	3.522	2.054–6.038	***
Program design characteristics			
Adolescents are involved in design/feedback	0.76	0.477–1.211	
Facility has strategy to involve adolescents^a^	0.906	0.743–1.107	
Privacy and confidentiality preserved^b^	1.151	1.026–1.292	*
Outreach and/or education is provided^c^	1.05	0.953–1.157	
RHC services discounted to adolescents	1.006	0.859–1.179	
Health facility characteristics			
Hours include evenings/weekend hours	1.115	1.025–1.212	**
Designated check-in and exam rooms available	0.968	0.777–1.205	
Waiting/exam rooms have print educational materials to appeal to adolescents	1.091	0.969–1.229	
Waiting/exam rooms have educational pictures to appeal to adolescents	1.101	1.002–1.209	*
Waiting/exam rooms have educational posters to appeal to adolescents	1.153	1.048–1.269	**

*β* Exponential estimates

Significance level: <0.0001***; <0.01**; <0.05*

N = 311

~ Model did not converge

^a^Facility has a strategy to involve adolescents in planning and in the provision of care

^b^Records are preserved to protect the privacy and confidentiality of adolescents’ personal medical records and health information

^c^Outreach and/or education provided in the community for young people

## Discussion

We sought to examine the density of RHC service availability and assess spatial patterns of these services in post-conflict Burundi. We also aimed to identify YFS practices associated with adolescents’ use of reproductive health services across health facility types. We found that RHC facilities were spatially clustered within three urban districts of Bujumbura Mairie province and in Isale, a district located in Bujumbura Rural province, with high densities of persistent violence acts (conflict, incidences, fatalities, and warring groups) nested within these districts. RHC facilities in Burundi show spatial patterning of distances, suggesting that most of the districts in the country (both rural and urban districts) have more than one facility located in close proximity to each other. Further, we found a stronger association between adolescents’ use of RHS and YFS practices. In particular, we found significant associations with facility characteristics (e.g., designated check-in and exam rooms) and programming characteristics (e.g., community outreach, privacy and confidentiality) and adolescents’ use of RHS across facility types.

Our analysis provides evidence that despite persistent violence in urban districts of Bujumbura Mairie, the administrative headquarters; adolescents’ use of RHC services was high, with use of services overlapping entirely with the density of violent locations. It’s possible therefore, that the recurring violence and adolescents’ use of services under such circumstances, although on one hand atypical, on the other, they have become the norm. These results corroborate the ideas of Jenkins [27], who suggested that for those existing in places of recurring political violence, “the extreme and the mundane are not necessarily alternative but simultaneous states of affairs that are lived with as a persistent existential contradiction”, for example, “living in a political ethos” or with respect to accessing basic needs (e.g., RHC services).

Another important finding was that the number of RHC facilities with no stock-outs of modern contraceptives in the past 3 months was high. Public centers had the highest availability of modern contraceptives than other centers and hospitals. It is possible, therefore, that a combination of government and international donors’ efforts to improve the availability of family planning methods at the service delivery point is achieving intended results. Burundi has made significant progress in improving the provision of RHC services and rights. For example, in 2008, the MoH through the National Reproductive Health Program (PNSR) initiated an approach aimed at improving the provision of YFS planning and RHC services to adolescents. Since then, policy and strategic documents have been adopted, among which, the National Population Policy Statement (2011), the National Health Development Plan and the revised National Sexual and Reproductive Health Strategy (2013–2015). Another example illustrating efforts by the Burundian government in tackling RHC services issues are reflected in key development frameworks, notably the “Vision Burundi 2025”, and the second Strategic Growth and Fight against Poverty (2012–2016) [18]. Burundi, therefore, provides an ideal opportunity to study how RHC services efforts made by the Burundian government to expand YFS have translated into practice at the facility level.

On the question of whether YFS practices are associated with adolescents’ use of RHS, this study found that RHC service facilities across Burundi vary in their provision of youth-friendly RHS to youth and young adults. We found that of the three YFS practices (facility, provider and programming factors), the majority of facilities are making their RHC facilities youth-friendly by designating rooms for adolescent check-in and examinations as well as making educational materials (e.g., pictures and print) on sensitive topics available in examination rooms.

Facilities are also very successful at incorporating outreach in communities and schools and in ensuring privacy and confidentiality of youth and young adults. These results seem to be consistent with previous studies [13, 28–30] which found privacy and confidentiality of young people during family planning and counselling as important determinants of use of services. The lack of youth engagement strategies and low cost/discounted RHC services to adolescents was apparent in both public and private RHC facilities. Public and religious facilities are not very effective at involving youth in the design of programs and in making decisions that affect them.

We also observed that most of the RHC facilities contracted under PBF have adopted the above program design practices (e.g., flexible hours) and practices to protect youth and young adults’ privacy and confidentiality, but not the other practices under study.

Notably, despite the existence of policy documents to build and enhance the capacity of RHC facilities and providers [19, 31], we found no evidence that provider training was statistically associated with adolescents’ use of RHC services. These results are in line with those of previous studies [32–34], and suggest a need for government commitment to provide tailored topical trainings to providers. These results are also likely to be related to the fact that the healthcare system in Burundi is afflicted by a lack of trained medical staff, resources to compensate them and a lack of modern equipment. This has led to an exodus of trained staff, leaving unskilled health workers to work beyond their capabilities in increasingly difficult circumstances.

This finding, while preliminary, suggests that a better understanding of the availability of youth-friendly RHS in the country could help program planners increase youth uptake of such services. It can thus be suggested that geography shapes both accessibility to RHS (high densities of RHC facilities) and can have an effect on an adolescent’s perception of the available services, “making contraceptives seem more common place and acceptable [22]”. Future studies on the current topic in post-conflict settings are therefore recommended.

Few studies to our knowledge have used both geospatial and non-spatial methods to explore utilization of RHC services among youth and young adults in post-conflict settings. The geospatial methods used in our study can be used to delineate RHC service availability, to estimate accessibility to these services, and to develop spatially targeted RHC services. Pre-conflict, governments can use the methods used to identify under-served or over-served areas, for example. In post-conflict settings, the methods can be used to identify and assess the availability and spatial patterns of RHC facilities to plan for targeted RHC service interventions, an important topic in the context of community recovery following civil unrest.

A limitation of this study is its cross-sectional nature, with data collected at a single point. But given the specific study objective, the study design is not a problem. The use of fine-grained geographic data from a census of facilities adds a broader, sector wide approach. Given that the census data for Burundi do not provide age specific population data, due to political reasons, it’s not possible to discern the population for young people; neither for those aged 10–24 years from the general population. This fact limits the interpretation of the data, particularly to the actual denominator of the population of interest. Finally, although the results are specific to the Burundi context, our mixed methods approach and results are generalizable to other countries and post-conflict settings.

## Conclusion

This study demonstrated how both geospatial and non-spatial methods can be used to (1) examine the density of RHS availability, (2) assess spatial patterns of these facilities, and (3) identify youth-friendly practices associated with adolescents’ use of services in post-conflict settings. The approaches used are generalizable to other post-conflict settings and to other types of interventions.
